# To engage or not engage: Early incentive motivation prevents symptoms of chronic post-stroke depression – A longitudinal study

**DOI:** 10.1016/j.nicl.2023.103360

**Published:** 2023-03-01

**Authors:** Janusz L. Koob, Shivakumar Viswanathan, Maike Mustin, Mia Mallick, Sebastian Krick, Gereon R. Fink, Christian Grefkes, Anne K. Rehme

**Affiliations:** a Department of Neurology, University Hospital Cologne, 50937 Cologne, Germany; b Institute of Neuroscience and Medicine, Cognitive Neuroscience (INM-3), Forschungszentrum Jülich, 52425 Juelich, Germany; c Department of Neurology, University Hospital Frankfurt, Goethe University Frankfurt, 60590 Frankfurt am Main, Germany

**Keywords:** Reward, Motor impairment, Grip force, Prognosis, PSD, Corticostriatal network lesions

## Abstract

**Background:**

Although post-stroke depression (PSD) is known to disrupt motor rehabilitation after stroke, PSD is often undertreated and its relationship with motor impairment remains poorly understood.

**Methods:**

In a longitudinal study design we investigated, which factors at the early post-acute stage may increase the risk for PSD symptoms. We were especially interested in whether interindividual differences in the motivational drive to engage in physically demanding tasks indicate PSD development in patients suffering from motor impairments. Accordingly, we used a monetary incentive grip force task where participants were asked to hold their grip force for high and low rewards at stake to maximize their monetary outcome. Individual grip force was normalized according to the maximal force prior to the experiment. Experimental data, depression, and motor impairment were assessed from 20 stroke patients (12 male; 7.7 ± 6.78 days post-stroke) with mild-to-moderate hand motor impairment and 24 age-matched healthy participants (12 male).

**Results:**

Both groups showed incentive motivation as indicated by stronger grip force for high versus low reward trials and the overall monetary outcome in the task. In stroke patients, severely impaired patients showed stronger incentive motivation, whereas early PSD symptoms were associated with reduced incentive motivation in the task. Larger lesions in corticostriatal tracts correlated with reduced incentive motivation. Importantly, chronic motivational deficits were preceded by initially reduced incentive motivation and larger corticostriatal lesions in the early stage post-stroke.

**Conclusions:**

More severe motor impairment motivates reward-dependent motor engagement, whereas PSD and corticostriatal lesions potentially disturb incentive motivational behavior, thereby increasing the risk of chronic motivational PSD symptoms. Acute interventions should address motivational aspects of behavior to improve motor rehabilitation post-stroke.

## Highlights

• Incentive motivated grip force on PSD development in motor impaired patients.
• More severe motor impairment after stroke increases incentive motivation.
• PSD and corticostriatal lesion may diminish incentive motivated behavior.
• Incentive motivation and corticostriatal lesion precede chronic motivation deficits.

## 1 Introduction

Stroke often leads to permanent disability, especially motor impairment (Virani et al., 2021). Patients suffering from stroke are also at risk of developing affective-depressive disorders, commonly described as post-stroke depression (PSD). To date, PSD often remains neglected, although it hinders rehabilitation and functional outcome (Medeiros et al., 2020, Robinson and Jorge, 2016). Depression includes heterogeneous symptoms such as emotional, cognitive, behavioral, and somatic disturbances, as well as motivational deficits or “apathy” (Hama et al., 2007a, Schmidt et al., 2008). Notably, stroke patients show a 3–4 times higher risk for depression compared to orthopedic patients or traumatic brain injury patients with comparable impairments or lesion volumes (Folstein et al., 1977, Robinson and Szetela, 1981). These findings suggest that PSD is a neurobiological consequence of the lesion rather than an adjustment to disability.

According to the monoamine hypothesis, depression may be caused by lesions of dopaminergic pathways in frontal and limbic structures constituting corticostriatal networks, which translate motivation into behavior (Loubinoux et al., 2012, Mayberg, 1997). Neuroimaging studies showed consistent evidence that corticostriatal networks subserve reward-related motor behavior, and corresponding lesions correlated with motivational deficits that worsen motor rehabilitation (Hama et al., 2007b, Rochat et al., 2013, Schmidt et al., 2008). The translation of motivation into physical action can be studied in experiments where motor effort is modeled by the response to an expected reward, referred to as *incentive motivation* (Berridge, 2004). Previous fMRI studies with healthy participants reported reward-dependent increases of neural activity in the ventral striatum (VS) that predicted (pre-)motor activity and the amount of motor effort during subsequent motor execution (Pessiglione et al., 2007, Schmidt et al., 2012). In stroke patients, a recent fMRI study found reduced VS activation in processing monetary reward feedback after motor performance in a motor pointing task suggesting impaired skill learning (Widmer et al., 2019). Likewise, studies including unipolar depressed patients showed impaired reward-learning processes and reduced monetary outcome (Admon and Pizzagalli, 2015, Cléry-Melin et al., 2019), especially when task-dependent effort was required (Cléry-Melin et al., 2011, Treadway et al., 2012). It remains unclear how motor and PSD symptoms, especially motivational deficits or apathy, early after stroke impact on incentive motivation to engage into motor effort and how incentive motivation influences the prognosis of depression.

The goal of our study was to investigate incentive-driven motor effort in stroke patients early post-stroke to predict chronic symptoms of PSD within the first-year. We examined patients with unilateral hand motor impairment and healthy participants using a grip force task adapted from the ‘classical’ monetary incentive delay task (Knutson et al., 2000, Knutson et al., 2005). Our participants exerted motor effort (i.e., grip force) over an extended 15 seconds (s) period to earn a monetary incentive of either 1cent (low reward) or 1€ (high reward). Using this task, we assessed how motor impairment and PSD affect incentive motivational behavior, defined as (i) greater grip force during high reward relative to low reward trials (‘reward effect’) and (ii) higher monetary outcome. Symptoms of PSD were reassessed >3 months post-stroke during their usual peak (Hackett and Pickles, 2014).

We hypothesized that both groups show incentive motivation leading to greater grip force in high relative to low reward conditions (Widmer et al., 2019). It is further assumed that early PSD or lesions of ventral and dorsal corticostriatal networks correlate with reduced incentive behavior. Regarding stroke rehabilitation, our task allowed us to evaluate whether motor impairment decreases or increases incentive motivation and whether early-stage incentive behavior prevents development of PSD symptoms at the chronic stage.

## 2 Methods and materials

### 2.1 Participants

Twenty-two right-handed first-ever stroke patients were recruited from the Department of Neurology, University Hospital Cologne, Germany, in the early days post-stroke. Inclusion criteria were: (i) first-ever ischemic or hemorrhagic stroke, (ii) unilateral upper-limb motor deficit with a residual motor function of the affected hand to hold a grip force sensor with 10 kPa minimum grip force, (iii) right-handedness. Exclusion criteria were: (i) bilateral stroke lesions, (ii) cognitive impairment, neglect or aphasia according to neurological examination, (iii) insufficient visual acuity (as assessed by clinical-neurological assessment and verified by the ability to read and follow the instructions on printed screenshots of the experiment), (iv) severe comorbid neurological or psychiatric disorders including preexisting depression, (v) any other motor impairments affecting upper limb motor function (e.g., rheumatoid arthritis, missing limbs). Two stroke patients were excluded due to incorrect performance or inability to finish the task. This resulted in a final sample of n = 20 stroke patients who were assessed on average 7.7 days post-stroke (SD = 6.78). One stroke patient received antidepressant medication post-stroke onset of 20mg fluoxetine per day. Twenty-four healthy right-handed participants, matched in age and gender, were included as control group.

The experiment was performed at the Department of Neurology, Cologne. Stroke patients were examined at bedside during hospitalization. All participants gave informed written consent in accordance with the local ethics committee and the Declaration of Helsinki (revised in 2008). Participants received their task-related monetary outcome in Euro after task completion. Controls additionally received an expense allowance of 15€.

### 2.2 Apparatus

The experiment was designed using PsychToolbox (Brainard, 1997, Kleiner et al., 2007) implemented in MATLAB2019a (The MathWorks Inc., Natick, MA, USA) on Windows. Visual stimuli were presented on a 19″-monitor. Grip force signals were acquired with a strain-gauge based isometric dynamometer with a 0–800 Newton (N) range (MLT004/ST, AdInstruments Ltd, New Zealand). Grip force signals were sampled at 1000 Hz using PowerLab 26T data acquisition system and LabChart software (AdInstruments Ltd, New Zealand). Acquired signals were continuously transferred to a custom-written MATLAB interface that used the grip force signals to update the visual display in real-time.

### 2.3 Experimental design

**Fig. 1:**
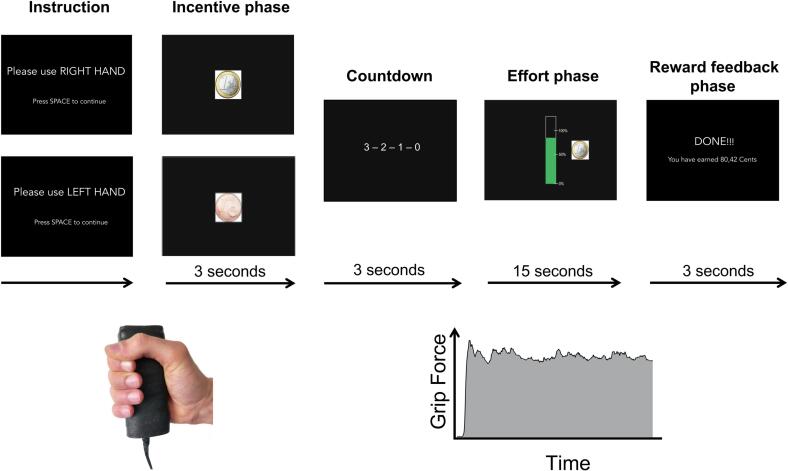
Study design of the monetary incentive grip force task. Prior to the experiment, participants were instructed to earn as much money as possible by using their grip force. Instruction: Trials started with instruction of either using the left or right hand in alternating order. Incentive phase (3s): The money at stake was displayed (1€ or 1ct). Countdown (3s): A countdown led to the effort phase. Effort phase (15s): A thermometer bar appeared that turned green relative to the exerted amount of grip force in real-time. The money at stake was presented throughout the effort phase. Reward feedback phase (3s): The amount of money was calculated based on the average grip force during the effort phase relative to the money at stake (1€ or 1ct) and displayed on the screen. Bottom left: An exemplary picture of a hand squeezing the grip force dynamometer. Bottom right: An illustration of an exemplary grip force curve during a trial. (For interpretation of the references to colour in this figure legend, the reader is referred to the web version of this article.)

The monetary incentive grip force task was designed following Pessiglione et al. (2007). Participants were seated in front of the screen in 1m distance, with both hands resting on their thighs and the grip force sensor in one hand. The grip force device was calibrated before the experiment to achieve a stable baseline. For calibration of the graphical representation, the maximum grip force was assessed by averaging the maximum force from three 3s intervals and used to represent 100% individual grip force. Calibration was done for the right and the left hand separately to control for differences in hand dominance or motor impairment. Each trial of the experiment was organized into an incentive phase, an effort phase, and a reward feedback phase (Fig. 1). Before the trial, participants were instructed to use either the left or right hand in alternating order between trials. During the incentive phase, subjects were informed about the money at stake by means of a picture showing either a one-cent or one-euro coin (3s). The picture resolution was 340x340 pixels with 8x8cm on the screen. The participants could earn the amount of money at stake using their grip force in the upcoming effort phase (15s). After a countdown of 3s, a thermometer bar appeared that turned green relative to the amount of grip force when pressing the device (Fig. 1). The thermometer level was updated in real-time based on the instantaneous grip force applied. In the reward feedback phase, the amount of money earned in each trial was presented in cents (3s). The monetary outcome was calculated based on the average grip force relative to the money at stake (1€ or 1ct) across the 15s effort phase. For example, a participant who squeezed on average 70% of his maximum grip force was rewarded with 70ct in a 1€-trial or 0.7ct in a 1-cent trial. A break was set after every five trials (30s). Prior to the experiment, participants were instructed to earn as much money as possible. The task consisted of a 2x2 factorial design with the factors HAND (levels: left, right) and REWARD (levels: low, high). The resulting four conditions were presented 10 times throughout the experiment, resulting in 40 identically structured trials. Using the right and left hand was required in an alternating order to control systematic hand fatigue effects. High and low reward trials were presented in a pseudo-random order with <4 equal reward trials in a row to prevent habituation.

### 2.4 Behavioral assessments

To assess motor deficits, we used the Jebsen-Taylor Hand-Function Test (JTT) (Jebsen et al., 1969), which evaluates the ability to perform hand movements of everyday life (e.g., picking up small items, lifting cans) using reaction time (in s). We calculated an index score by using the time of the unaffected relative to the affected hand in percent to control for individual differences in motor speed. This means that a lower index (<100%) represents greater motor impairment. Additionally, the National Institutes of Health Stroke Scale (NIH-SS) (Brott et al., 1989), which is an observer rating to quantify neurological impairments in a global disability score, was obtained from the medical records. For quantifying depressive symptoms, we used the Montgomery-Åsberg Depression Rating Scale (MADRS) interview, which includes ten items to describe depression (Herrmann et al., 1998, Montgomery and Asberg, 1979). Importantly, each item must be answered based on several questions according to published guidelines (Williams and Kobak, 2008). As depression constitutes a heterogeneous syndrome and we were particularly interested in incentive motivational behavior, a *motivational* score was formed based on detailed interview questions for item 7 representing apathy, difficulties of getting started and perform daily activities and item 8 representing loss of interest and reduced ability to react with adequate emotion to circumstances. Previous studies showed that principal component analyses in elderly and stroke patients yielded a factor structure where these two items loaded on one factor named apathy/retardation/anhedonia (Farner et al., 2009, Parker et al., 2003). Furthermore, we tested the inter-rater reliability of items 7 and 8 included in a motivational score based on an independent rating of seven clinical psychologists, which resulted in substantial agreement (kappa = 0.625) (Landis and Koch, 1977).

#### 2.4.1 Follow-up assessment

For follow-up assessment, patients were invited for a MADRS interview >3 months post-stroke (mean = 5.77 months, SD = 2.74). This time was referred to as the chronic stage when PSD symptoms peak (Hackett and Pickles, 2014). Data from n = 15 patients (75%) could be obtained. Reasons for dropouts were second strokes (10%), inability to perform the interview (5%), or disapproval (10%).

### 2.5 Grip force pre-processing

Grip force data were processed using MATLAB R2019a. Prior to the analysis, small negative grip force signals (in N) resulting from subtle oscillations were set to zero. Then, data were standardized using the maximum grip force per trial to yield relative values independent of the level of motor performance/impairment. No further filtering or smoothing was applied.

Data from patients with left-hemispheric stroke (45%) were flipped to assure that the affected hand corresponded to the left hand in all patients (Rehme et al., 2011a). Consequently, the same proportion of data was flipped in age- and gender-matched healthy controls. Thus, after flipping, the right hand did not correspond to the dominant hand in all participants and findings allow no conclusion on hand dominance effects. Please note that the left or right hand of the healthy participants is termed ‘affected’ or ‘unaffected’ compared to the respective hand of stroke patients.

We determined two parameters to depict incentive motivational behavior in the grip force task. First, the reward effect was calculated by subtracting the mean relative grip force in low from high reward trials to assess physical engagement for monetary incentives. Second, the monetary outcome was the accumulated win based on the overall motor effort at the end of the experiment. There was no fixed upper limit of monetary gains. Whereas the reward effect depends on economical strategies to maximize monetary outcome by preserving the grip force for high versus low reward trials, the overall monetary outcome rather reflects the mere motivation for motor effort driven by monetary incentives.

Furthermore, we analyzed whether motor fatigue during the course of the experiment affects incentive motivational behavior. Therefore, we computed the difference in relative grip force between the first and second half of all trials.

### 2.6 Structural lesion analysis

For n = 14 patients (70%), anatomical MRI scans obtained during the clinical routine were available to map the stroke lesion based on diffusion-weighted imaging (DWI; TR = 4076ms, TE = 95ms, 22–24 axial slices, voxel size = 1.8x2.99x6mm^3^) and fluid-attenuated inversion recovery images (FLAIR; TR = 6000ms, TE = 100ms, 36–40 axial slices, voxel size = 1.38x1.1x4mm^3^). Lesion maps were manually drawn onto DWI images showing the extent of the ischemic lesion. These images were spatially co-registered to Montreal Neurological Institute (MNI) standard space, resampled to 1x1x1mm voxel size, and normalized using unified segmentation with masked lesions (Ashburner and Friston, 2005) in SPM12 (https://www.fil.ion.ucl.ac.uk/spm/software/spm12/) as implemented in MATLAB 2019a. Finally, lesion maps were binarized for further analyses using FMRIB Software Library (FSL, https://fsl.fmrib.ox.ac.uk/fsl/fslwiki). To test neuroanatomical correlates of depression, we generated binary maps of the online published diffusion tensor imaging (DTI) atlas based on data from 1065 healthy participants depicting the course of the ventral and dorsal corticostriatal tracts (Yeh et al., 2018). The binary atlas map was co-registered with the lesion maps. As the raw atlas tract showed small disruptions after normalization which was inappropriate for lesion-symptom mapping, the tract was smoothed using a 6mm Gaussian smoothing kernel in SPM12 (Fig. 4A). Percent overlaps between individual lesion maps and the corticostriatal atlas map were computed using SPM12 and FSL.

Twelve out of 15 patients (85%) examined in the follow-up assessment had anatomical MRI scans from the acute stage available for PSD prognosis.

### 2.7 Statistical analysis

Generally, group differences in age, JTT-index, and monetary outcome were analyzed using one-way ANOVA in SPSS version 26 (IBM Corp, Armonk, NY, USA). In case of ordinal scaled variables such as MADRS score and motivational score, Mann-Whitney U-rank tests were used. Significant effect sizes were determined by partial eta-squared (η_p_^2^) as provided by SPSS. For the experimental task, relative mean grip force and fatigue effects were analyzed using repeated-measures ANOVA and Bonferroni-corrected post-hoc t-tests with the within-subject factors HAND (affected vs. unaffected hand) and REWARD (high vs. low reward) and the between-subject factor GROUP including stroke patients and controls.

Correlations between two interval-scaled variables including JTT-index, reward effect, monetary outcome, and relative lesion volume were analyzed using Pearson correlations (R_P_). For ordinal-scaled parameters including global MADRS and motivational score, Spearman correlations (R_Sp_) were computed. All correlation analyses were two-sided (p < 0.05), except for correlations between incentive motivation and depression parameters based on previous evidence (Admon and Pizzagalli, 2015, Cléry-Melin et al., 2019, Cléry-Melin et al., 2011, Rochat et al., 2013, Schmidt et al., 2008, Treadway et al., 2012) (one-sided p < 0.05). We computed the following sets of correlation analyses with false-discovery-rate (FDR) correction applied for multiple testing (Benjamini and Hochberg, 1995). First, within the group of stroke patients, correlation analyses were computed between the incentive motivation parameters (i.e., reward effect, monetary outcome) and JTT-index, NIH-SS, global MADRS and motivational score. Second, for PSD prognosis at the chronic stage, correlations between initial behavioral scores including global MADRS, motivational score, and the reward effect and the follow-up assessment of global MADRS and motivational score were computed. For lesion-symptom analyses, we correlated the percent of lesion overlap in the ventral and dorsal corticostriatal tracts with the global MADRS or motivational score at initial and follow-up assessment as well as with the reward effect. Lastly, to investigate whether multiple predictors explain a higher amount of variance in post-stroke motivational deficits in the chronic stage, we computed stepwise linear regression models. MADRS motivational score at chronic stage was included as dependent variable and reward effect and lesion overlap in ventral and dorsal corticostriatal tracts as three different predictor variables.

## 3 Results

### 3.1 Demographics and group characteristics

Stroke patients (n = 20) were on average 71.55 (SD = 10.71) years old and control subjects (n = 24) were 66.96 (SD = 7.84) years old. This difference did not reach statistical significance (F_1,42_ = 2.688, p = 0.109). Furthermore, groups did not differ in the proportion of sex (χ^2^_1_ = 0.440, p = 0.507). 12 female participants were included in the control group, 8 females in the patient group, as well as 12 male participants in both the control and patient group. Regarding the MADRS score and the motivational score, stroke patients showed on average more depressive symptoms (M = 11.20, SD = 6.83) than controls (M = 2.50, SD = 2.27; U = 36.500, p < 0.001, η_p_^2^ = 0.527), as well as stroke patients came with higher motivational deficits (M = 2.15, SD = 1.84) than controls (M = 0.33, SD = 0.56; U = 85.500, p < 0.001, η_p_^2^ = 0.349). Similarly, groups significantly differed in their motor impairment score (JTT index; stroke patients: M = 37.35, SD = 30.01, controls: M = 98.04, SD = 10.41; F_1,42_ = 86.074, p < 0.001, η_p_^2^ = 0.672). Stroke patients were assessed on average 7.7 days after stroke onset (SD = 6.78), with nine patients had left-hemispheric lesions (11 patients right-hemispheric), and 18 patients suffered from ischemic strokes (two patients had hemorrhagic strokes). One stroke patient received antidepressant medication post-stroke onset of 20mg fluoxetine per day. See Table 1 for all demographics and group characteristics.

**Table 1:** Overview of the demographic and clinical characteristics of the sample.

	Stroke patients n = 20 mean (SD)	Controls n = 24 mean (SD)	Statistical test
Demographics			
Sex (f:m)	8:12	12:12	χ^2^_1_=0.440, p=0.507
Mean age (years) (±SD)	71.55 (10.71)	66.96 (7.84)	F_1,42_=2.688, p=0.109
Days post-stroke (±SD)	7.7 (6.78)		
Lesion side (left:right)	9:11		
Stroke (ischemic:hemorrhagic)	18:2		
Motor impairment			
JTT-index (%) (±SD)	37.35 (30.01)	98.04 (10.41)	F_1,42_=86.074, p<0.001**, η_p_^2^=0.672
Global impairment			
NIH-SS	8.48 (4.63)		
Depressive symptoms			
MADRS global score _early_ (±SD)	11.20 (6.83)	2.50 (2.27)	U=36.500, p<0.001**, η_p_^2^= 0.527
Motivational score _early_ (±SD)	2.15 (1.84)	0.33 (0.56)	U=85.500, p<0.001**, η_p_^2^=0.349
MADRS global score _follow-up_ (±SD)	7.93 (5.86)		
Motivational score _follow-up_ (±SD)	2.86 (2.83)		


JTT: Jebsen-Taylor Hand Function Test, NIH-SS: National Institutes of Health Stroke Scale, MADRS: Montgomery-Åsberg Depression Rating Scale, χ^2^: chi square, η_p_^2^: Partial eta-squared effect size, U: Mann-Whitney U. One patient received antidepressive medication of 20mg fluoxetine/day. MADRS scores in the early stage post-stroke were: no depression (n = 7), mild depression (n = 11), moderate depression (n = 2). MADRS scores in the chronic stage were: no depression (n = 10), mild depression (n = 4), moderate depression (n = 1) (Herrmann et al., 1998).

### 3.2 Monetary incentive grip force task

**Fig. 2:**
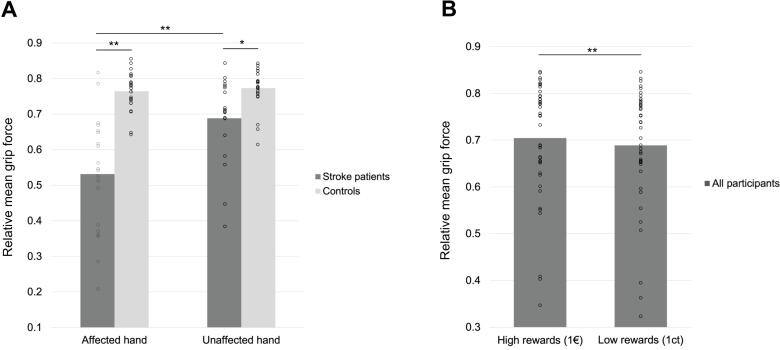
Monetary incentive grip force task. A: Relative mean grip force of stroke patients and controls in the monetary incentive grip force task per hand. B: Relative mean grip force of all participants in the high compared to low reward condition. (**= p < 0.01, *= p < 0.05).

The repeated-measures ANOVA yielded a significant interaction between HAND and GROUP (F_1,42_ = 30.030, p < 0.001, η_p_^2^ = 0.417) (Fig. 2A). Overall, stroke patients showed lower relative grip force as compared to controls. Likewise, there was a significant main effect of GROUP (F_1,42_ = 31.519, p < 0.001, η_p_^2^ = 0.429) and HAND (F_1,42_ = 37.783, p < 0.001, η_p_^2^ = 0.474). There was a main effect of REWARD, that is, all participants showed greater grip force for high compared to low reward trials (F_1,42_ = 17.332, p < 0.001, η_p_^2^ = 0.292) (Fig. 2B). However, there was neither an interaction between GROUP and REWARD (F_1,42_ = 0.155, p = 0.696), nor a difference between groups in their monetary outcome (F_1,42_ = 2.238, p = 0.142), so the magnitude of incentive motivation parameters was not statistically different between groups. Importantly, there was no significant correlation between the reward effect and the monetary outcome, neither in the overall sample (R_P_ = -0.010, p = 0.947), nor in the subgroup of stroke patients (R_P_ = -0.316, p = 0.175), reflecting two different aspects of incentive motivation.

The repeated-measures ANOVA of experimental fatigue yielded a significant main effect of HAND (F_1,42_ = 7.102, p = 0.011, η_p_^2^ = 0.145). That is, the affected hand showed stronger muscular fatigue over the course of the experiment. Furthermore, there was no statistically significant interaction between HAND and GROUP (F_1,42_ = 2.931, p = 0.094), nor a significant main effect of REWARD (F_1,42_ = 2.900, p = 0.096), yet significance trends could be noticed. Importantly, there was neither an interaction effect between REWARD and GROUP (_F1,42_ = 0.386, p = 0.538), nor a main effect of GROUP (F_1,42_ = 0.260, p = 0.613). The experimental variables of the monetary incentive grip force task are summarized in Supplementary Table S1.

### 3.3 Stroke subgroup analysis

**Fig. 3:**
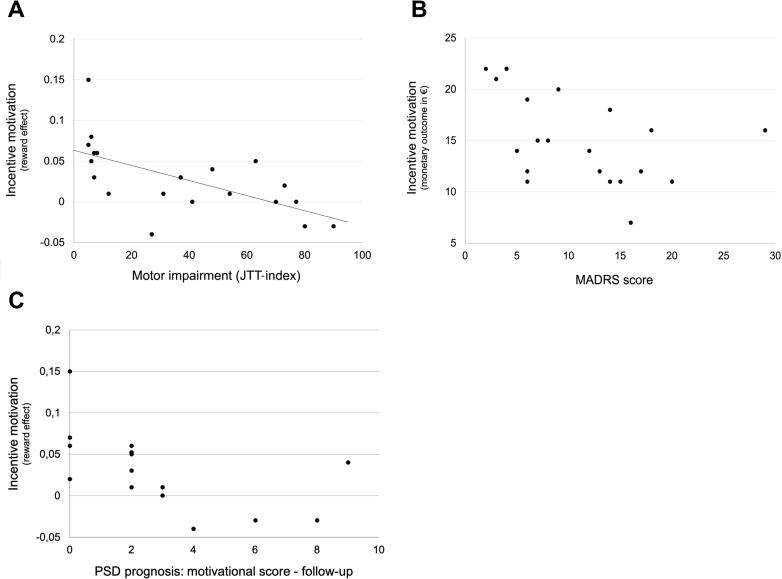
Relationships with incentive motivational behavior. A: Negative correlation between the reward effect in the monetary incentive grip force task and the motor impairment (JTT-index) in stroke patients (n = 20; R_P_ = -0.630, p = 0.024). Lower JTT-index scores indicate higher motor impairment. More severely impaired stroke patients showed a higher reward effect. B: Negative correlation between the monetary outcome in the task and the global MADRS score in stroke patients (n = 20; R_Sp_ = -0.490, p = 0.037). More depressed stroke patients earned less money in the task. C: Prognosis of PSD. Negative correlation between the reward effect in the monetary incentive grip force task and the motivational score at follow-up (n = 15; R_Sp_ = -0.718, p = 0.015). Stroke patients who showed higher reward effects in the monetary grip force task were less prone to motivational deficits at the chronic stage.

The correlation analysis in the group of stroke patients showed that the reward effect correlated negatively with the JTT-index (R_P_ = -0.630, p = 0.024, FDR-corrected) (Fig. 3A). This means that patients with more severe hand motor impairment exhibit greater incentive motivation as assessed by stronger grip force at high versus low reward trials. Likewise, the NIH-SS correlated positively with the reward effect (R_Sp_ = 0.592, p = 0.024, FDR-corrected), which indicates that more severe global impairment is associated with greater incentive motivation.

The global MADRS correlated negatively with the overall monetary outcome (R_Sp_ = -0.490, p = 0.037, FDR-corrected) (Fig. 3B). That is, more depressed stroke patients earned less money in the experiment based on their overall motor performance than less depressed patients. Likewise, there was a non-significant trend between the global MADRS score and the reward effect (R_Sp_ = 0.372, p = 0.084, FDR-corrected). Interestingly, the MADRS score did not correlate with the JTT-index (R_Sp_ = -0.343, p = 0.158, FDR-corrected), thus, in our sample of stroke patients with residual hand motor function, depressive symptoms were not associated with motor impairment early post-stroke. Yet there was a non-significant trend for a correlation between MADRS and NIH-SS indicating greater depression among more severely impaired patients (R_Sp_ = 0.435, p = 0.073, FDR-corrected).

#### 3.3.1 PSD prognosis

In stroke patients, there was a negative correlation between the experimental reward effect and the motivational score at follow-up (R_Sp_ = -0.718, p = 0.015, FDR-corrected) (Fig. 3C). That is, patients with initially higher incentive motivation showed less motivational deficits at the chronic stage. There were no correlations between initial and follow-up global MADRS scores (R_Sp_ = -0.369, p = 0.352, FDR-corrected), motivational scores (R_Sp_ = 0.009, p = 0.974, FDR-corrected), and between the reward effect and the follow-up MADRS (R_Sp_ = -0.069, p = 1.000, FDR-corrected).

#### 3.3.2 Structural lesion analysis

**Fig. 4:**
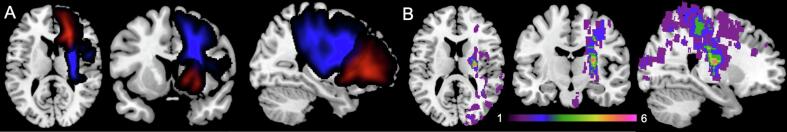
Structural lesion analysis. A: Anatomical DTI tract template of the corticostriatal tracts (Yeh et al., 2018) with a 6mm Gaussian smoothing. Red: ventral corticostriatal tract. Blue: dorsal corticostriatal tract. B: Lesion overlap map. The highest overlap was at the level of the internal capsule. (For interpretation of the references to colour in this figure legend, the reader is referred to the web version of this article.)

The anatomical DTI template of the dorsal and ventral corticostriatal tracts (Yeh et al., 2018) are shown in Fig. 4A. Stroke patients had the highest lesion overlap at the level of the internal capsule (30%) (Fig. 4B). The percent of the lesion in the ventral corticostriatal tract correlated negatively with the reward effect (R_P_ = -0.711, p = 0.012, FDR-corrected). This indicates that patients with more extended lesions of the ventral corticostriatal tract were less likely to be motivated by reward to engage in physical activity. Furthermore, the follow-up motivational score correlated positively with lesions in the dorsal tract (R_Sp_ = 0.684, p = 0.042, FDR-corrected), which suggests that patients with more extended lesions of the dorsal tract were more likely to develop motivational deficits at the chronic stage. There were no correlations between initial MADRS or motivational score and corticostriatal lesions (p > 0.207).

To test whether multiple predictors improve the prognosis of motivational deficits in the chronic stage, we additionally performed stepwise regression models. Overall, reward effect, lesion overlap of dorsal and ventral corticostriatal tract did not explain more variance in stepwise executed regressions than the reward effect and the dorsal corticostriatal tract alone (coefficients of correlation in regression models ranged from R = 0.484 to R = 0.516).

## 4 Discussion

Incentive motivation early after stroke is influenced by different factors, including the degree of motor impairment, damage in corticostriatal networks, and PSD. Importantly, incentive motivation was increased in patients with more severe motor impairments and decreased in patients with greater PSD symptoms. In our sample, early incentive motivation served as one prognostic factor of reduced risk for motivational deficits or apathy at the chronic stage, whereas patients with lesions of dorsal and ventral corticostriatal tracts showed reduced incentive motivation and a higher risk for motivational deficits at the chronic stage. This is one of the first longitudinal studies showing that behavioral parameters of incentive motivation preceded motivational deficits at the chronic stage post-stroke. An overview of the main study results is provided as Supplementary Fig. S2.

### 4.1 Monetary incentive motivation and motor impairment

Both groups showed similar incentive motivational behavior in the task. Another study investigating reward-modulated motor performance post-stroke reported similar findings (Widmer et al., 2019). Interestingly, we found that stroke patients with greater motor impairment maximized their monetary outcome by preserving their grip force for high versus low reward trials. Importantly, as the relative grip force is higher despite limited resources, this economical behavior may reflect a general psychological phenomenon of the underlying motivational state of the patients rather than an effect of the lesion and concomitant motor impairment. This motivational state might represent an adaptive coping strategy considering that task difficulty of holding one’s grip force is increased given restricted physical abilities (Raghavan, 2015).

Importantly, post-stroke fatigue is a prevalent condition in the acute and chronic stages after stroke and can negatively affect rehabilitation outcome (de Groot et al., 2003, Nadarajah and Goh, 2015). Generally, it can be differentiated between motor fatigue, which indicates a muscular exhaustion after physical effort, and chronic fatigue as a strong feeling of tiredness and lethargy or apathy, which typically does not improve with rest and represents motivational fatigue (de Doncker et al., 2018, de Groot et al., 2003, Tseng et al., 2010). In our sample, muscular fatigue was stronger for the affected hand, probably because recruitment of motor units was reduced due to the ischemic lesion to motor tracts. Notably, as there were no interactions and effects of the reward conditions on experimental fatigue, we assume that incentive motivation was not driven by fatigue.

### 4.2 Monetary incentive motivation and PSD

Early symptoms of PSD showed a correlational trend for a reduced reward effect in the monetary grip force task. Nevertheless, considering the monetary outcome, patients with greater depression earned significantly less money in the task. Of note, the potential monetary gain had no upper limit to motivate subjects with normal reward processing to try to further increase their grip force. Hence, our finding of less monetary outcome in more depressed patients suggests that they were less motivated to generally engage in reward-related physical effort, independent of the money at stake. This is in line with previous studies including unipolar depressed or apathetic stroke patients (Cléry-Melin et al., 2011, Rochat et al., 2013, Schmidt et al., 2008). Nevertheless, this is the first study showing that depressive symptoms correlate with incentive motivational behavior early after stroke.

#### 4.2.1 Prognosis of PSD

Various clinical studies reported a higher risk for PSD at chronic stages 3–12 months post-stroke (Medeiros et al., 2020, Robinson and Jorge, 2016). A novel finding of our study is that the reward effect as a measure of incentive motivation had predictive power for later motivational PSD symptoms or apathy. Here, increased motivation to engage in incentive-driven motor activity may potentially prevent later motivational deficits. Interestingly, other studies found an antidepressant effect of motor activity in patients (Cuijpers et al., 2007, Loubinoux et al., 2012, Yuan et al., 2015). According to the reinforcement model (Lewinsohn, 1974), depression results from a lack of positive reinforcement based on reduced behavioral activity, which in turn may increase the experience of ‘learned helplessness’, which is also triggered by stroke (Seligman, 1972). Thus, in terms of a vulnerability-stress model, the stroke lesion may induce a vulnerability that can lead, driven by psychological factors including loss of positive reinforcement and learned helplessness, into PSD. Due to the relatively small sample size, results must be considered as preliminary, and effects may be underpowered. However, given the difficulty to assess early-stage stroke patients with a high risk of later drop-outs, the results reveal important aspects of the symptomatology of PSD. Please also note that control subjects did not receive a follow-up assessment in our study and thus, due to a lack of comparability, our findings of incentive motivation as a prognostic factor for later motivational deficits cannot assuredly be assigned to a patient-specific phenomenon. However, as healthy control participants were included only in absence of any neurological or psychiatric disorders, we assumed no change in depressive symptoms or motivational deficits over time, so control subjects were assessed only once (Rehme et al., 2011b).

Importantly, chronic MADRS scores were not associated with initial MADRS scores suggesting that the monetary incentive grip force task represents a covert but more specific measure of the underlying motivational state and for prognosis of chronic motivational deficits. As our task required patients with residual hand motor function and consent, patients with different impairments might develop greater PSD or less incentive motivation. Furthermore, our sample comprised patients with relatively mild depressive symptoms and good recovery thereof. In comparison to our findings, other studies found that PSD during the first-year post-stroke depend on earlier MADRS scores (Fuentes et al., 2009, Mikami et al., 2021). This difference may partly result from different sample sizes, but also different examination stages or impairment levels. For example, Mikami et al. (2021) assessed 48 patients within the first 6 weeks post-stroke with lower impairment severity (NIH-SS: mean 3.9) compared to our sample (NIH-SS: mean 8.48) assessed on average 7.7 days post-stroke. Thus, chronic PSD may be reliably predicted by the presence of depressive symptoms in the subacute stage rather than the early stage post-stroke.

#### 4.2.2 Structural lesion sites and PSD

We found that ventral corticostriatal tract lesions were specifically associated with impaired incentive motivation in task performance, whereas lesions of the dorsal corticostriatal tract were associated with prognosis of motivational PSD symptoms at later stages. Corticostriatal projections have been shown to be involved in goal-directed behaviors, including the motivation and cognitions leading to these actions (Haber, 2016). As the *dorsal* tract connects higher-order sensorimotor areas with dorsal striatum (Alexander and Crutcher, 1990, Draganski et al., 2008, Haber and Knutson, 2010), our finding corroborates that this circuit plays a vital role for motivated behaviors and that apathy following stroke may impair motor rehabilitation (Hama et al., 2007b, Rochat et al., 2013, Schmidt et al., 2008). Interestingly, a multicenter study showed that the antidepressant selective serotonin-reuptake-inhibitor fluoxetine improves motor performance independent of changes in PSD symptoms (Chollet et al., 2011). The *ventral* tract connects primarily ventro-medial prefrontal and orbitofrontal cortices with the VS, including nucleus accumbens, that has been shown to be involved in translating motivation into action and reward learning (Haber and Knutson, 2010). It has been frequently reported that reduced fMRI activity in dorsolateral prefrontal cortex and VS correlates with reduced incentive motivation in unipolar depressed patients (Cléry-Melin et al., 2011, Knutson et al., 2008, Robinson et al., 2012). Additionally, incentive motivation has been shown to be reduced by apathy post-stroke, resulting from damage to bilateral basal ganglia including VS (Rochat et al., 2013, Schmidt et al., 2008). Our preliminary findings of a prognosis for motivational deficits suggest – in line with extensive evidence from structural and functional neuroimaging studies – that incentive motivation requires an intact reward system as prerequisite for motor recovery and prevention of motivational PSD symptoms.

### 4.3 Conclusion

This study demonstrates for the first time that early-stage incentive motivation might reduce the risk for motivational PSD symptoms or apathy at the chronic stage. Thus, early motivation post-stroke may be a key target for interventions to improve rehabilitation and individual life quality while reducing public healthcare costs (Robinson and Jorge, 2016, Widmer et al., 2022). Additional studies of early-stage stroke patients are warranted to identify further PSD predictors and determine valuable treatment approaches.

## CRediT authorship contribution statement

**Janusz L. Koob:** Data curation, Formal analysis, Writing – original draft, Writing – review & editing. **Shivakumar Viswanathan:** Conceptualization, Formal analysis, Writing – review & editing. **Maike Mustin:** Data curation, Formal analysis, Writing – review & editing. **Mia Mallick:** Data curation. **Sebastian Krick:** Data curation. **Gereon R. Fink:** Writing – review & editing. **Christian Grefkes:** Conceptualization, Funding acquisition, Supervision, Resources, Writing – review & editing. **Anne K. Rehme:** Conceptualization, Funding acquisition, Data curation, Investigation, Formal analysis, Supervision, Writing – original draft, Writing – review & editing.

## Declaration of Competing Interest

The authors declare that they have no known competing financial interests or personal relationships that could have appeared to influence the work reported in this paper.

## Data Availability

Data will be made available on request.
