# Supplementary material for: To engage or not engage: Early incentive motivation prevents symptoms of chronic post-stroke depression – A longitudinal study
Source: Neuroimage Clin. 2023 Mar 1;37:103360. doi: 10.1016/j.nicl.2023.103360 (PMC10009723; doi:10.1016/j.nicl.2023.103360)
Supplement: Supplementary data 1 [file mmc1.pdf]

Supplementary table S1: Sample characteristics in grip force performance in the monetary incentive grip force task.

	Stroke patients n = 20 mean (SD)	Controls n = 24 mean (SD)	Statistical test
Mean relative grip force (±SD)	0.61 (0.13)	0.77 (0.05)	$F_{1,42}$ = 31.519, p < 0.001**, η_p^2 = 0.429
Mean relative grip force $_{AffH}$ (±SD)	0.53 (0.16)	0.76 (0.05)	$F_{1,42}$ = 43.774, p < 0.001**, η_p^2 = 0.510
Mean relative grip force $_{UnaffH}$ (±SD)	0.69 (0.12)	0.77 (0.06)	$F_{1,42}$ = 9.919, p = 0.003**, η_p^2 = 0.191
Mean absolute grip force (in N) (±SD)	118.79 (81.30)	215.73 (54.29)	$F_{1,42}$ = 22.261, p < 0.001**, η_p^2 = 0.346
Mean absolute grip force$_{AffH}$ (in N) (±SD)	68.28 (82.00)	213.06 (63.00)	$F_{1,42}$ = 43.838, p < 0.001**, η_p^2 = 0.511
Mean absolute grip force$_{UnaffH}$ (in N) (±SD)	169.30 (91.22)	218.39 (50.21)	$F_{1,42}$ = 5.111, p = 0.029*, η_p^2 = 0.108
Reward effect (±SD)	0.03 (0.04)	0.03 (0.05)	$F_{1,42}$ = 0.155, p = 0.696
Reward effect $_{AffH}$ (±SD)	0.01 (0.03)	0.02 (0.03)	$F_{1,42}$ = 0.823, p = 0.370
Reward effect $_{UnaffH}$ (±SD)	0.01 (0.03)	0.01 (0.03)	$F_{1,42}$ = 0.136, p = 0.714
Fatigue effect (±SD)	-0.015 (0.045)	-0.009 (0.033)	$F_{1,42}$ = 0.260, p = 0.613
Fatigue effect $_{AffH}$ (±SD)	-0.057 (0.118)	-0.024 (0.069)	$F_{1,42}$ = 1.340, p = 0.254
Fatigue effect $_{UnaffH}$ (±SD)	-0.002 (0.091)	-0.012 (0.068)	$F_{1,42}$ = 0.154, p = 0.697
Monetary outcome (in €) (±SD)	14.95 (4.25)	16.83 (4.08)	$F_{1,42}$ = 2.238, p = 0.142

AffH: affected hand, UnaffH: unaffected hand.

As an additional motor impairment indication, we performed the ‘Hand’ and ‘Sensation’ subtests of the upper limb extremity functions of the Fugl-Meyer Assessment (FMA) (Fugl-Meyer, Jääskö, Leyman, Olsson & Steglind, 1975). Stroke patients performed significantly worse in the FMA (M = 21.5, SD = 5.07) compared to controls (M = 26, SD = 0) ($F_{1,42}$ = 18.974, p < 0.001**, η_p^2 = 0.490).

An additional questionnaire was the German version of the Aspiration Index (AI) of current life goals. The AI assesses the priority of specific current life goals and needs as well as the perceived probability of achieving them in the dimensions of monetary wealth, personal growth, social relationships, and physical health (Kasser & Ryan, 1993). Between stroke patients and controls, the dimensions of the Aspiration Index (AI) of momentary values and needs showed no significant differences (all p > 0.098). Within the stroke patients subgroup, the AI showed a correlation between the expectation of physical health in the future and MADRS score (R_{Sp} = -0.646, p = 0.012, FDR-corrected) as well as between the importance of health and the JTT index (R_{Sp} = 0.513, p = 0.033, FDR-corrected). Hence, higher depression and greater motor impairment reflected less confidence or less importance in physical health. Furthermore, there was a negative correlation between the JTT-index and the perceived probability of achieving monetary wealth (R_{Sp} = -0.543, p = 0.032, FDR-corrected). That is the confidence into monetary wealth as a behavioral marker of

motivation was greater in those patients with stronger motor deficits. Likewise, the reward effect correlated positively with expected monetary wealth ($R_{Sp} = 0.511$, $p = 0.025$, FDR corrected).

Supplementary figure S2: Overview of the main study results. Green arrow lines indicate a supportive association, red arrow lines indicate a detrimental association, and the dotted black line indicates no significant correlation. In the early stage post-stroke, reduced incentive motivation in the monetary grip force task was related to less motor and global impairment, a greater percentage of corticostriatal tract lesions, and more pronounced PSD symptoms. Initially higher incentive motivation in the task predicted a decreased risk of motivational deficits in PSD symptoms in the chronic stage post-stroke. Furthermore, greater damage of the dorsal tract predicted later PSD symptoms. PSD symptoms in the early stage post-stroke had no predictive value for PSD symptoms in the chronic stage.

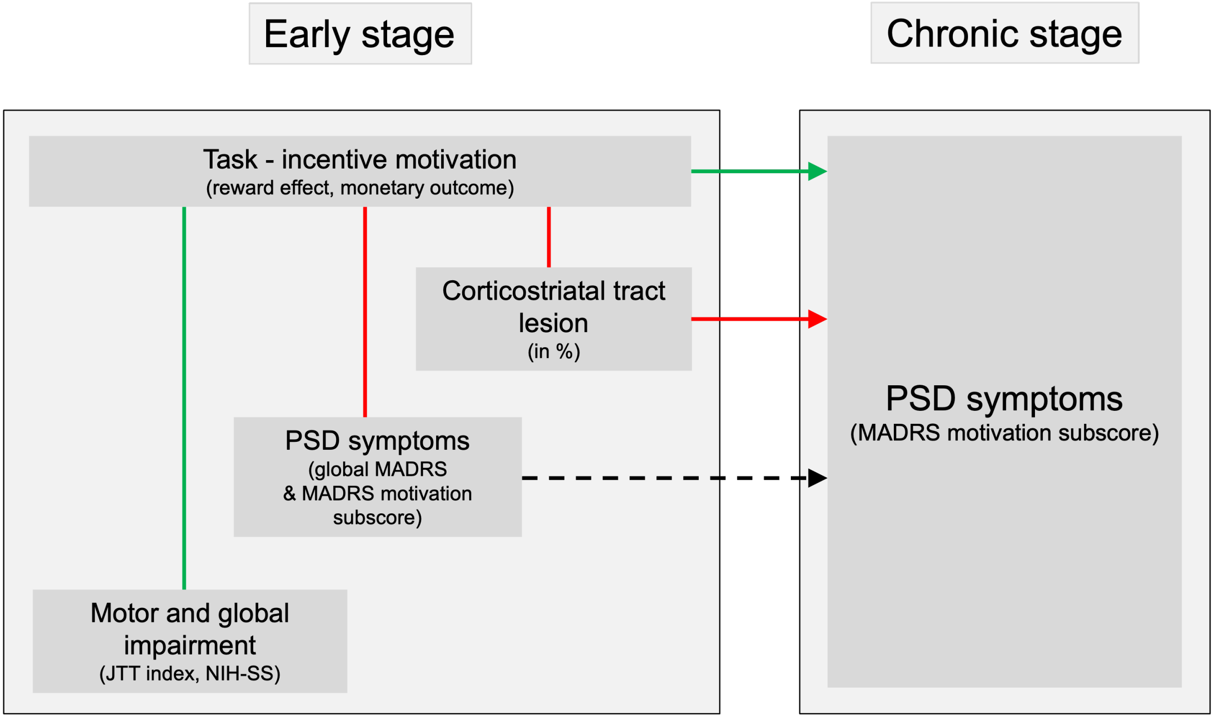

Supplementary references

Fugl-Meyer, A. R., Jääskö L., Leyman I., Olsson S., & Steglind S. (1975): A method for evaluation of physical performance. *Scandinavian Journal of Rehabilitation Medicine, 7*(1), 13-31.

Kasser T., & Ryan R.M. (1993): A Dark Side of the American Dream: Correlates of Financial Success as a Central Life Aspiration. *Journal of personality and social psychology, 65*(2): 410–422. doi:10.1037/0022-3514.65.2.410
